# Truncating *SLC5A7* mutations underlie a spectrum of dominant hereditary motor neuropathies

**DOI:** 10.1212/NXG.0000000000000222

**Published:** 2018-03-23

**Authors:** Claire G. Salter, Danique Beijer, Holly Hardy, Katy E.S. Barwick, Matthew Bower, Ines Mademan, Peter De Jonghe, Tine Deconinck, Mark A. Russell, Meriel M. McEntagart, Barry A. Chioza, Randy D. Blakely, John K. Chilton, Jan De Bleecker, Jonathan Baets, Emma L. Baple, David Walk, Andrew H. Crosby

**Affiliations:** From the RILD Wellcome Wolfson Centre (C.G.S., H.H., K.E.S.B., M.A.R., B.A.C., J.K.C., E.L.B., A.H.C.), Royal Devon & Exeter NHS Foundation Trust, Exeter; Wessex Clinical Genetics Service (C.G.S.), Princess Anne Hospital, Southampton, United Kingdom; Neurogenetics Group (D.B., I.M., P.D.J., T.D., J.B.), Center for Molecular Neurology, VIB; Laboratory of Neuromuscular Pathology (D.B., I.M., P.D.J., T.D., J.B.), Institute Born-Bunge, University of Antwerp; Department of Neurology (M.B., D.W.), University of Minnesota, Minneapolis, MN; Department of Neurology (P.D.J., J.B.), Neuromuscular Reference Centre, Antwerp University Hospital, Antwerpen, Belgium; Clinical Genetics (M.M.M.), St. George's University of London, London, United Kingdom; Biomedical Science (R.D.B.), Florida Atlantic University, Jupiter Campus, FL; and Department of Neurology (J.D.B.), University Hospital Ghent, Ghent, Belgium; Peninsula Clinical Genetics Service (E.L.B.), Royal Devon and Exeter Hospital, Exeter, United Kingdom.

## Abstract

**Objective:**

To identify the genetic cause of disease in 2 previously unreported families with forms of distal hereditary motor neuropathies (dHMNs).

**Methods:**

The first family comprises individuals affected by dHMN type V, which lacks the cardinal clinical feature of vocal cord paralysis characteristic of dHMN-VII observed in the second family. Next-generation sequencing was performed on the proband of each family. Variants were annotated and filtered, initially focusing on genes associated with neuropathy. Candidate variants were further investigated and confirmed by dideoxy sequence analysis and cosegregation studies. Thorough patient phenotyping was completed, comprising clinical history, examination, and neurologic investigation.

**Results:**

dHMNs are a heterogeneous group of peripheral motor neuron disorders characterized by length-dependent neuropathy and progressive distal limb muscle weakness and wasting. We previously reported a dominant-negative frameshift mutation located in the concluding exon of the *SLC5A7* gene encoding the choline transporter (CHT), leading to protein truncation, as the likely cause of dominantly-inherited dHMN-VII in an extended UK family. In this study, our genetic studies identified distinct heterozygous frameshift mutations located in the last coding exon of *SLC5A7*, predicted to result in the truncation of the CHT C-terminus, as the likely cause of the condition in each family.

**Conclusions:**

This study corroborates C-terminal CHT truncation as a cause of autosomal dominant dHMN, confirming upper limb predominating over lower limb involvement, and broadening the clinical spectrum arising from CHT malfunction.

Distal hereditary motor neuropathies (dHMNs) are a clinically and genetically heterogeneous group of diseases characterized by distal lower motor neuron dysfunction without major upper motor neuron or sensory involvement. In 1993, Harding classified dHMN into 7 subtypes (dHMN-I to VII) according to the mode of inheritance and clinical features, since which classification has evolved further through improved knowledge of molecular pathogenesis. Previously, we identified a frameshift (c.1497delG) mutation in *SLC5A7* (NM_021815.2), encoding the hemicholinium-3 (HC-3)-sensitive Na+/Cl-dependent, presynaptic choline transporter (CHT; NP_068587.1) critical for normal neuromuscular junction (NMJ) signaling, as the cause of a dominantly-inherited motor neuron disease (dHMN-VII).^1^ This variant resulted in a translational frameshift of CHT, causing premature termination (p.Lys499Asnfs*13) and protein truncation, with near-complete deletion of the cytoplasmic C-terminus. Transporter assays revealed significant reductions in HC-3-sensitive choline uptake due to the p.Lys499Asnfs*13 mutation, consistent with a dominant-negative mode of action.^1^ Here, we report additional dominant truncating CHT mutations in 2 unrelated families for which individuals were referred initially with diagnoses of amyotrophic lateral sclerosis (ALS) based on a progressive pure motor neurogenic disorder with hyperreflexia, and Charcot-Marie-Tooth (CMT) type II based on distinct and progressive atrophy of the hand muscles and gait difficulties. This clinical presentation is then compared with the recently described phenotype arising from recessively acting CHT mutations, consisting of a spectrum of congenital myasthenic syndrome (CMS) disorders, associated with biallelic *SLC5A7* mutations*.*

## Methods

### Clinical studies

Detailed pedigree information was collated, and thorough neurologic examination was performed in the proband, mother, and 3 maternal uncles/aunts of family A, and the proband, mother, and sister of family B. Nerve conduction studies and/or needle EMG was performed in 4 individuals in family A and the proband and sister of family B.

### Genetic studies

DNA was extracted from peripheral blood samples obtained from family members with informed consent. DNA for next-generation sequencing performed on the proband of each family was enriched for target regions using the Illumina TruSight One-Sequence capture panel (family A) or the Nextera Rapid Capture Expanded Exome kit (62 Mb) (family B), with the prepared library sequenced on an Illumina HiSeq 2500 and annotation and variant filtering using the Clinical Sequence Analyzer and Miner (WuXi NextCODE). Cosegregation of each variant was confirmed using dideoxy sequence analysis.

Information on these exomes, including a list of genes covered, can be accessed at support.illumina.com/downloads/trusight_one_sequencing_panel_product_file.html, and illumina.com/documents/products/datasheets/datasheet_nextera_rapid_capture_exome.pdf, respectively.

### Standard protocol approvals, registrations, and patient consents

Approval was obtained from the Institutional Review Board of the University of Minnesota and the Ethical Standards Committee of Antwerp Hospital Centre for clinical research activities performed in this investigation. Written informed consent was obtained from all research participants.

## Results

### Clinical findings

#### Family A, individual 1: (A:IV:1)

**Figure 1:**
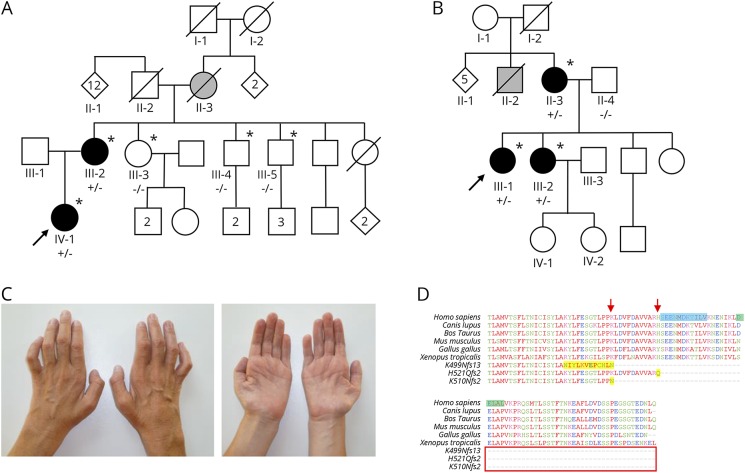
Family pedigrees, clinical photographs, and multispecies alignment showing the effect of the 3 reported mutations (A–B) Pedigrees of families A and B showing affected (black), unaffected (white), and possibly affected (grey) individuals. Those clinically investigated are indicated by * and those who had genetic testing are indicated by the test result, where (+/−) represents individuals heterozygous for the *SLC5A7* mutation and (−/−) represents individuals homozygous for wild type. (C) Hands of individual B:III:1 showing pronounced atrophy of the intrinsic hand muscles. (D) Species amino acid sequence alignment of the CHT C-terminal region, depicting the outcomes of the K499Nfs13, H521Qfs2, and K510Nfs2 alterations (arrows indicate the position of polypeptide truncation, and additional aberrant amino acids are highlighted in yellow). The primary endocytic motif (SEENMDKTILV-1° Motif) is highlighted in blue (Ser522 to Val532), and the secondary endocytic motif (DELAL-2° Motif) is highlighted in green (Asp540 and Leu544).

A 27-year-old woman was referred with a diagnosis of possible ALS after a 1- to 2-year history of progressive weakness in the hands without sensory symptoms, dysarthria, or dysphagia. Family history at the time was reported to be negative for neurologic disease. There were no cognitive deficits, and cranial nerve examination, including bulbar examination and articulation, was normal. Perception of touch, vibration, and pinprick were unremarkable. Motor examination demonstrated marked atrophy in intrinsic hand muscles bilaterally, normal muscle tone, and no fasciculations. Manual muscle testing demonstrated full strength except in distal muscles (table 1). Reflexes were brisk throughout, with vertical spread in the upper limbs but without clonus or Babinski signs. Nerve conduction studies demonstrated markedly attenuated median and peroneal compound muscle action potentials (CMAPs) with normal conduction velocities and no focal motor conduction block, normal ulnar and tibial motor conduction studies, and normal sensory nerve action potential amplitudes (table 2). Needle EMG demonstrated fibrillation potentials, reduced interference patterns, and high-amplitude long-duration motor unit potentials in distal muscles of upper and lower limbs. A serum study of ganglioside GM-1 antibodies was negative. Hexosaminidase levels, *SOD1* sequencing, CSF cell count, and protein and glucose levels were normal (figure 1A).

**Table 1:**
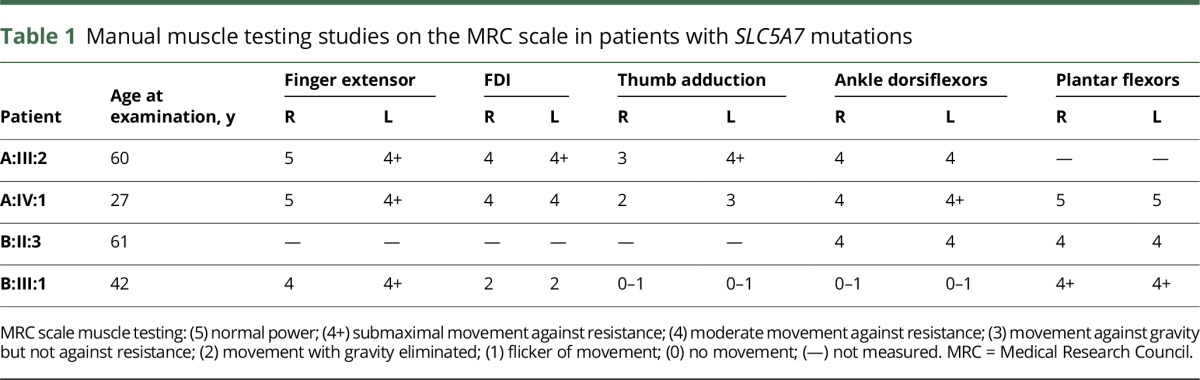
Manual muscle testing studies on the MRC scale in patients with *SLC5A7* mutations

**Table 2:**
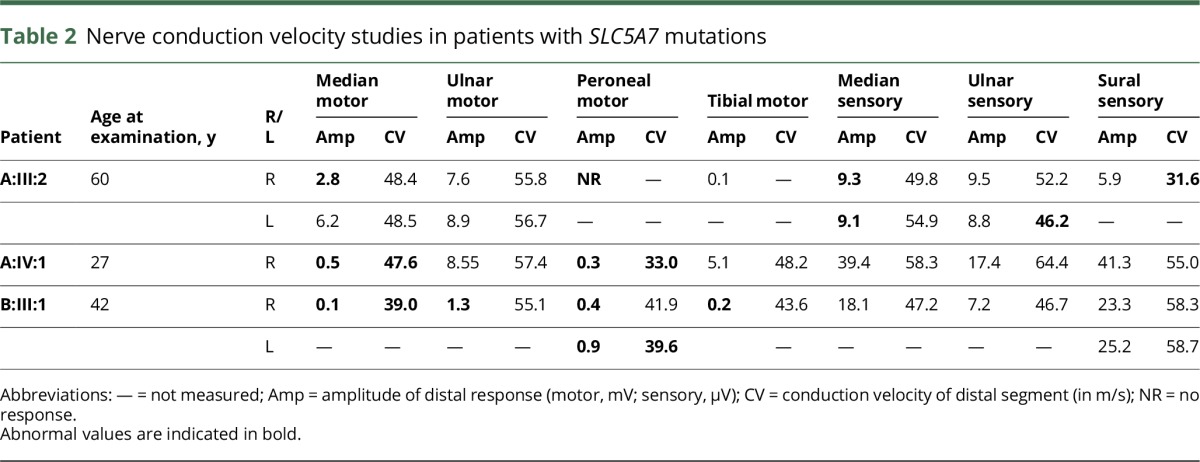
Nerve conduction velocity studies in patients with *SLC5A7* mutations

#### Family A, individual 2: (A:III:2)

Twelve years later, the proband's mother presented at age 60 years with a 3-year history of gradually progressive weakness of ankle plantarflexion bilaterally. There was no history of sensory symptoms, weakness in the upper limbs or proximal lower limbs, dysphagia, or dysarthria. There were no cognitive deficits, and cranial nerve examination, including bulbar examination and articulation, was normal. Sensory examination demonstrated normal perception of touch and vibration and an equivocal reduction in pinprick perception in the toes. Motor examination demonstrated mild atrophy of intrinsic muscles of the hands and feet. Muscle tone was normal. No fasciculations were noted. Manual muscle testing demonstrated full strength except for distal muscles (table 1). Reflexes were brisk with vertical spread in the upper limbs and normal in the lower limbs without clonus or Babinski signs. Nerve conduction studies demonstrated attenuated median, peroneal, and tibial CMAPs with normal conduction velocities and no focal motor conduction block, normal ulnar motor conduction studies, and normal or borderline findings on orthodromic sensory nerve conduction studies (table 2). Needle EMG demonstrated fibrillation potentials, reduced interference patterns, and high-amplitude long-duration motor unit potentials in distal muscles. Serum studies for ganglioside GM-1 and Lyme antibodies were negative. Vitamin B12, copper levels, CSF cell count, and protein and glucose levels were normal. Laryngoscopy demonstrated normal vocal cord function (figure 1A).

These patients have been followed for 14 and 2 years, respectively, with no clinically significant change in examination or function, except for the development of median entrapment at the wrist, confirmed with electrodiagnostic study, in the proband.

The mother of the proband has 4 living siblings, 3 of whom have provided written consent to examination and genetic evaluation, and 2 of these to electrodiagnostic testing, under a protocol approved by the University of Minnesota Institutional Review Board. None of these 3 had clinical or electrodiagnostic evidence of motor neuropathy, and they were wild type and had not inherited have the *SLC5A7* variant identified in the proband and her mother. The patients also reported that their deceased maternal grandmother/mother had hand deformities late in life that were believed to be due to arthritis (A:II:3). No other family members are known to have symptoms or signs of neuromuscular disease (figure 1A).

#### Family B, individual 1: (B:III:1)

A 41-year-old woman was first seen by a neurologist at the age of 24 years with a diagnosis of possible CMT type II. Prior history reveals subtle difficulties with fine motor skills in the hands in primary school, at that time without gait difficulties. Progressive difficulty running combined with frequent ankle sprains became apparent at the age of 15 years. Distal weakness in both upper and lower limbs progressed, and she used ankle-foot orthoses from the age of 30 years. Gait autonomy is currently well preserved with walking aids. Despite relatively pronounced atrophy of the intrinsic hand muscles, she was a nurse until the age of 39 years and is currently a head nurse with more administrative tasks. The patient denies sensory symptoms but recognizes a slight hoarseness that is more pronounced with fatigue or upper respiratory tract infection (figure 1B).

On examination, the patient had no cognitive deficits. Cranial nerve examination was normal aside from hoarseness (dysphonia), without dysarthria. Touch, pinprick, and vibration sense and proprioception were normal. Motor examination demonstrated bilateral pes cavus, hammer toes, and marked distal atrophy in the lower limbs, most notable in the anterior compartment. In addition, there was pronounced atrophy of the intrinsic hand muscles (figure 1C). Manual muscle testing demonstrated full strength in all proximal muscles and abnormal distal strength in the upper limbs (table 1). Reflexes were markedly brisk, with exception of the Achilles tendon reflex, which was absent. Hoffmann sign was present on the right and absent on the left. Plantar reflexes were mute in severely paretic feet. The patient had a steppage gait. Nerve conduction studies demonstrated a pure motor axonal neuropathy without conduction blocks (table 2). Routine concentric needle EMG showed chronic neurogenic changes, and single-fiber EMG revealed mild to moderate abnormalities of neuromuscular transmission in both orbicularis oculi and extensor digitorum communis muscles. Formal speech analysis and laryngostroboscopy confirmed the presence of a left paramedian vocal fold paralysis.

#### Family B, individual 2 (B:III:2)

The older sister of the proband presented with a history of progressive atrophy in the hand muscles and bilateral pes cavus starting in her early twenties. At age 46 years, she had distinct atrophy of the abductor pollicis brevis muscles and, to a lesser extent, atrophy of the intrinsic hand and foot muscles. Like her sister, there was no sensory involvement. There was no distinct gait difficulty aside from slight eversion of the feet. Manual muscle testing demonstrated full strength in the proximal muscles and minimally reduced strength in foot dorsiflexion, without a manifest foot drop. Reflexes in the upper limbs were normal; patellar and ankle reflexes were bilaterally reduced; and plantar responses were flexor. Nerve conduction studies and needle EMG were consistent with a pure motor axonal polyneuropathy (figure 1B).

#### Family B, individual 3 (B:II:3)

The mother of the proband, 61 years of age at examination, demonstrated atrophy of intrinsic hand muscles, most notably in the abductor pollicis brevis. This patient also presented with atrophy of the foot muscles and the distal third of the legs, as well as bilateral pes cavus. Manual muscle testing demonstrated full strength in the proximal muscles and decreased strength in distal lower extremity muscles (table 1). The patellar reflexes were reduced and ankle reflexes absent. Other reflexes were normal including plantar responses. No nerve conduction studies or needle EMG was performed. The clinical examination, however, is compatible with a distal motor neuropathy with pronounced involvement of the hands and is similar to the phenotype seen in her daughters, albeit milder (figure 1B).

The mother of the proband has 6 siblings, one of whom was reported to have gait difficulty. While no further information is available on this individual (1B:II:2), this may indicate the presence of other affected family members. The 5 remaining siblings and maternal grandparents are reportedly unaffected. The proband has 2 unaffected siblings, and the children of both affected sister and unaffected brother show no symptoms.

### Genetic findings

Initial analysis of variants in genes with known associations with neuropathy identified a potentially deleterious heterozygous sequence variant in *SLC5A7* in each family: a heterozygous 2-bp deletion in family A (NM_021815.2**:** c.1561_1562delCA) and a 1-bp deletion in family B (NM_021815.2: c.1528delA). The variant was confirmed by dideoxy sequence analysis and found to cosegregate appropriately for an autosomal dominant disorder in each family.

**Figure 2:**
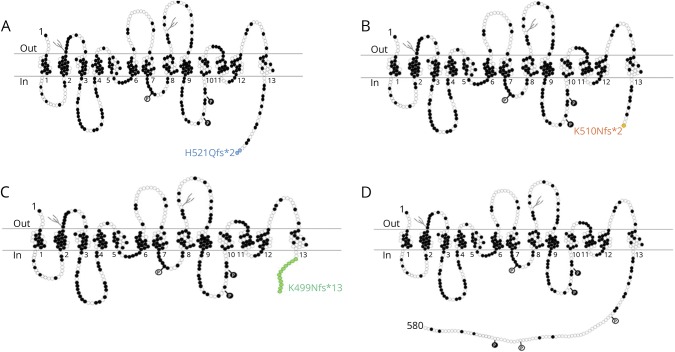
Schematic displaying the 3 described CHT mutant proteins alongside wild type molecule (Adapted from reference 2, using Microsoft Powerpoint Software) Schematic indicating the transmembrane architecture and C-terminal truncation of both sequence variants identified in the current study alongside the previously published dHMN-VII-associated CHT sequence variant (A: H521Qfs*2; B: K510Nfs*2; C: K499Nfs*13; D: wild type). Dark circles represent amino acids that are conserved across human CHT, mouse Cht1, and the C. elgans CHT ortholog. Potential protein kinase A phosphorylation sites are indicated by light circles containing a letter P, and potential protein kinase C phosphorylation sites are indicated by dark circles containing a letter P. Potential N-glycosylation sites are indicated by gray “tree-like” structures.

Wild-type CHT comprises 580 amino acids predicted to encompass 13 transmembrane domains, with an extracellular NH_2_-terminus and an intracellular COOH-terminus.^2,3^ The deletion in family A is predicted to result in a substitution of glutamine for histidine at residue 521, followed by a premature stop codon, resulting in a truncation of the COOH terminus (p.His521Glnfs*2). The variant in family B is predicted to result in a translational frameshift, substituting lysine at amino acid position 510 for asparagine, and going on to encode 1 additional aberrant amino acid before a premature termination in translation (p.Lys510Asnfs*2). Similar to the *SLC5A7* c.1497delG/p.Lys499Asnfs*13 variant previously found to be responsible for dHMN-VII,^1^ the c.1561-1562delCA and c.1528delA variants identified here are predicted to result in premature truncation of the protein. The family A (c.1561_1562delCA) variant results in CHT truncation by 59 amino acids with the inclusion of 1 aberrant glutamine residue; p.His521Glnfs*2, whereas the family B variant (c.1528delA) results in the same premature stop codon as the previously described dHMN-VII (c.1497delG) variant, resulting in truncation of the last 82 amino acids (p.Lys510Asnfs*2). A schematic to aid visualization of the predicted outcome of each variant on the polypeptide sequence of CHT is shown in figure 2, A-D.

Online mutation analysis tools including PROVEAN (provean.jcvi.org/index.php) and MutationTaster (www.mutationtaster.org/) predicted both previously identified c.1497delG^1^ and newly identified c.1561-1562delCA and c.1528delA variants to have a deleterious effect on CHT function (PROVEAN: −55.034, −14.155, and −55.034; MutationTaster: 1, 0.99 and 1, respectively). Conservation analysis indicates that the C-terminal region affected corresponds to a region of the molecule that is highly conserved across many species (figure 1D). TMHMM analysis (www.cbs.dtu.dk/services/TMHMM/) for the in silico definition of transmembrane domain architecture predicts that all 3 variants (figure 2, A-D) will profoundly affect polypeptide topology at the 3′ CHT terminus (data not shown).

Notably, all variants including the previously identified c.1497delG (p.Lys499Asnfs*13) frameshift mutation responsible for dHMN-VII^1^ and the newly identified c.1561-1562delCA (p.His521Glnfs*2) and c.1528delA (p.Lys510Asnfs*2) mutations are located in the last exon of the gene and will likely escape nonsense-mediated mRNA decay leading to the generation of a truncated polypeptide product. Consistent with each being pathogenic, all 3 frameshift variants are absent from the gnomAD database of ∼138,000 controls (gnomad.broadinstitute.org/).

## Discussion

We describe 2 families with novel C-terminal *SLC5A7* mutations predicted to result in production of a truncated CHT protein: family A (proband and mother) with a dHMN phenotype associated with a cosegregating p.His521Gln*fs2 mutation and family B (proband, mother, and sister) also with a dHMN phenotype associated with a cosegregating p.Lys510Asnfs*2 mutation. These findings are in line with the p.Lys499Asnfs*13 truncating CHT mutation that we previously reported in a large family with multiple individuals affected by dHMN-VII. In this original family, transporter assays showed a significant reduction in mutant transporter activity compared with wild type (WT) likely due to a dominant-negative mutational mechanism.^1^

Several clinical findings are of note in the families reported here. First, the absence of vocal cord paralysis in family A represents a notable clinical difference when compared with the proband of family B and the previously described family. Second, we find it notable that both patients in family A report a relatively abrupt progression of symptoms over a few years, followed by clinical stability. Progression is more gradual in family B. Electrodiagnostic findings indicate that denervation, which is accentuated distally in both patients, is chronic and likely preceded initial symptoms. On presentation, the findings of family A's proband raised concern about possible ALS because of definite lower motor neurone signs in association with hyperreflexia and reflex spread, which also holds true for family B's proband. Thus, several features were atypical or cautionary for a diagnosis of sporadic ALS, including the relatively early age at onset, arguably slow progression, and the absence of spasticity, clonus, or extensor plantar responses, features that would represent unequivocal evidence of upper motor neurone dysfunction. Given this, the fact that hyperreflexia was not seen in all patients, and the lack of a compelling mechanism for upper motor neurone involvement, the significance of the hyperreflexia seen in some patients is unknown. Thus, our patients demonstrate that a diagnosis of ALS should be made with caution in individuals presenting with distal weakness and atrophy and hyperreflexia without more compelling evidence of upper motor neurone disease.

This phenotypic variability is again mirrored in the absence of hoarseness in both mother and sister in family B, who also show a milder overall phenotype and later age at onset. In both families, several affected individuals display pronounced and early involvement of hand intrinsic muscles. This could be a useful clinical clue to help orient diagnosis toward *SLC5A7*. Upper limb predominance is also a known feature of dHMN subtypes caused by *BSCL2* and *GARS* mutations.^4^

The CHT cytoplasmic C-terminus that is truncated by the p.His521Gln*fs2 and p.Lys510Asnfs*2 mutations reported here and the p.Lys499Asnfs*13 reported previously,^1^ contain sequences known to drive constitutive endocytosis. A primary endocytic motif (SEENMDKTILV-1° Motif) between CHT amino acids Ser522 to Val532 contains a dileucine-type motif and supports dynamin-dependent endocytosis.^5^ A secondary motif (DELAL-2° Motif) is also present between Asp540 and Leu544 of the C-tail that supports enhanced surface expression of CHT but does not exhibit a dominant capacity for surface trafficking.^5^ The CHT truncating mutations described in all 3 families result in the complete elimination of SEENMDKTILV-1° and DELAL-2° motifs (figure 1D), suggesting that these mutations may lead to impaired choline uptake via the loss of dynamin-dependent endocytosis. Of interest, inspection of online genome sequence databases via gnomAD (gnomad.broadinstitute.org/) identifies 3 rare heterozygous CHT truncating variants (present in 11 individuals) in the last exon of *SLC5A7*. Notably, all 3 affect residues located after the primary endocytic motif between residues Ser522 and Val532 (at Lys538, Arg548, and Thr558). This may indicate that variants resulting in CHT truncation before the endocytic motif may be the primary driver for dHMN-related phenotypes. This may occur via 2 possible mechanisms. First, the endocytotic sequences are used to bud proteins for transport between the endoplasmic reticulum and golgi apparatus, allowing them to enter trafficking pathways to the nerve terminus. Failure or abnormalities in this process, due to a lack of these motifs, may therefore result in cell soma accumulation and reduction in synaptic membrane CHT and choline uptake. Second, endocytotic sequences are likely to have a central role in the binding of endocytic adaptors (e.g., AP2/AP3) in routing CHT to synaptic vesicles.^6^ The presence of CHT in the synaptic vesicles provides a rapid supply of CHT at the presynaptic nerve terminus, which is essential to ensure that the choline is efficiently recaptured from the rapidly hydrolysed ACh in the synaptic cleft. Defects in this mechanism would allow choline to diffuse away from the synaptic cleft and may result in an overall reduction in choline uptake. However, it remains unclear whether the 3 individuals listed in online databases gnomAD and ExAC, with an apparent heterozygous variant in the last exon of *SLC5A7* that would likely eliminate the primary endocytic motif (Tyr466 and Arg382), may display features of dHMN or whether CHT truncation at these residues may be variably or nonpenetrant.

Before the discovery of impaired CHT activity, NMJ dysfunction had not previously been implicated in the dHMNs. However, it is a well recognized cause of CMS, a genetically heterogeneous group of neuromuscular disorders, which is known to result from mutations in genes that encode presynaptic and postsynaptic proteins at the NMJ such as choline acetyltransferase (*CHAT*), ACh receptors, receptor-associated protein of the synapse (*RAPSN*), and downstream of kinase 7 (*DOK7*).^7–11^ More recently, homozygous and compound heterozygous mutations in *SLC5A7* have been reported in a spectrum of CMS disorders ranging from arthrogryposis to neonatal presentation with hypotonia, fatigable muscle weakness, and respiratory insufficiency with recurrent apneas. These include missense mutations located upstream of the cytoplasmic tail and a single N-terminal nonsense mutation (p.Ile42*)^12^ likely to result in nonsense mediated decay. The likely result of these mutations is the production of exclusively mutant CHT polypeptide(s) with intact C-terminal and endocytic motifs.^12,13^ dHMN types V and VII do not share apparent phenotypic features with CMS other than vocal cord involvement. The cardinal feature of muscle fatigability is absent in both affected families as are ophthalmoparesis, ptosis, and bulbar weakness; furthermore, unlike CMS, features do not appear until adulthood. No signs of neuropathy have been reported in autosomal recessive *SLC5A7* CMS, although it is possible that these features might develop with time. In patient B:III.1, single-fiber EMG revealed mild to moderately increased jitter measurements in the absence of impulse blocking in both Musculus orbicularis oculi and the Musculus extensor digitorum communis. These findings are difficult to interpret in this clinical context because a neurogenic process such as dHMN is known to cause secondary alterations in NMJ function, although we would expect this phenomenon mainly in a clinically affected distal muscle and less so in the Musculus orbicularis oculi. This single observation is insufficient to confirm a primary defect of NMJ transmission in patients with *SLC5A7*-linked dHMN.

The very different phenotypes seen in dominant and recessive *SLC5A7*-associated CHT disorders are likely to result from specific and differing outcomes particular to each class of mutation on CHT activity and trafficking of the molecule.^1,9^ Although the C-terminal truncating (dominant-negative) mutations greatly reduce CHT functionality, their deleterious impact on overall CHT activity appears to be less than in the case of pathogenic missense (recessive) variants, which have compounding deleterious outcomes on both CHT trafficking and specific activity.^12,13^

Specific clinical outcomes may also reflect the specific functional impact on the NMJs that vary across human muscle fiber types, placing differing demands on the CHT protein. The common “en plaque” NMJ has a single innervation to each muscle fiber, whereas “en grappe” NMJs have diffuse, multiterminal connections. These “en grappe” NMJs innervate tonic muscle fibers, which can continuously release ACh by almost an order of magnitude longer than twitch terminals. They are present only in a small number of human muscles, namely the extraocular, stapedius, tensor tympani, laryngeal, and tongue.^14^ CHT, and its central role in choline recycling, may be of particular importance in this sustained, high-frequency ACh signaling and therefore may result in fatigability in these muscle groups.

Here, we provide additional convincing genetic and functional evidence to confirm dominant mutations leading to impaired CHT function as a cause of dHMN phenotypes and demonstrate that an absence of vocal cord involvement in patients with dHMN with upper limb involvement predominating over lower limb does not preclude *SLC5A7* CHT involvement.

## Glossary

AChR: ACh receptor
ALS: amyotrophic lateral sclerosis
CHT: choline transporter
CMAP: compound muscle action potential
CMS: congenital myasthenic syndrome
CMT: Charcot-Marie-Tooth
dHMN: distal hereditary motor neuropathy
NMJ: neuromuscular junction
